# Surface Activation and Pretreatments for Biocompatible Metals and Alloys Used in Biomedical Applications

**DOI:** 10.1155/2019/3806504

**Published:** 2019-06-02

**Authors:** Vivian Huynh, Ngan K. Ngo, Teresa D. Golden

**Affiliations:** Department of Chemistry, University of North Texas, 1155 Union Circle #30507, Denton, TX 76203, USA

## Abstract

To improve the biocompatibility of medical implants, a chemical composition of bone-like material (e.g., hydroxyapatite) can be deposited on the surface of various substrates. When hydroxyapatite is deposited on surfaces of orthopedic implants, several parameters must be addressed including the need of rapid bone ingrowth, high mechanical stability, corrosion resistance, biocompatibility, and osseointegration induction. However, the deposition process can fail due to poor adhesion of the hydroxyapatite coating to the metallic substrate. Increasing adhesion by enhancing chemical bonding and minimizing biocoating degradation can be achieved through surface activation and pretreatment techniques. Surface activation can increase the adhesion of the biocoating to implants, providing protection in the biological environment and restricting the leaching of metal ions in vivo. This review covers the main surface activation and pretreatment techniques for substrates such as titanium and its alloys, stainless steel, magnesium alloys, and CoCrMo alloys. Alkaline, acidic, and anodizing techniques and their effects on bioapatite deposition are discussed for each of the substrates. Other chemical treatment and combination techniques are covered when used for certain materials. For titanium, the surface pretreatments improve the thickness of the TiO_2_ passive layer, improving adhesion and bonding of the hydroxyapatite coating. To reduce corrosion and wear rates on the surface of stainless steel, different surface modifications enhance the bonding between the bioapatite coatings and the substrate. The use of surface modifications also improves the morphology of hydroxyapatite coatings on magnesium surfaces and limits the concentration of magnesium ions released into the body. Surface treatment of CoCrMo alloys also decreased the concentration of harmful ions released in vivo. The literature covered in this review is for pretreated surfaces which then undergo deposition of hydroxyapatite using electrodeposition or other wet deposition techniques and mainly limited to the years 2000-2019.

## 1. Introduction

Hydroxyapatite (HAp) coatings have been studied for the field of orthopedics and dentistry due to its engineered similarity to the human bone matrix. Its inorganic matrix can be synthetically created from various simulated body fluid (SBF) solutions, commonly known as Hank's, Ringer's, and Kokubo's solution [1–3]. Tadashi Kokubo established a SBF solution in the 1990s to show the similarity between in vitro and in vivo behavior of specific glass-ceramic compositions [1]. Much research has been dedicated to modifying the SBF solutions to improve the quality of bioactivity and biocompatibility of the coatings [4, 5]. Recently, Leena et al. have developed a method for the acceleration of HAp synthesis process from more than 24 to 3 hrs [6]. For implant applications, metallic substrates are coated with HAp not only to minimize direct metal-body fluid contact, but to improve biocompatibility and bioactivity for the new formation of bone [7, 8]. The HAp coating provides a barrier between the releases of harmful elements from the metal substrate into the body and also reduces the friction coefficient from the implant and its surroundings [9].

Even though HAp is biocompatible, its poor adhesion properties to the substrate make it difficult for coating load-bearing devices. In vivo tests of HAp coatings have shown lack of bonding strength to the metal substrate or resorption into the body [4, 5, 7]. Different electrochemical deposition techniques, such as electrophoretic, pulse potential, and direct potential, have been implemented to improve the adhesion strength and its long-term reliability [7, 10]. However, adhesion strength is also affected by different surface activation techniques. Surface activation techniques are processes in which the substrate is modified via pretreatment steps in order to change the surface topography, the chemical composition, and structure of the oxide layer and to form new surface features [9]. Surface activation can increase the adhesion of HAp on implants by altering the chemical bonds on the substrate and minimizing biocoating degradation. Activating the surface provides protection against in vivo body fluid and restrains the penetration of metal ions into organisms, reducing the corrosion of the implant (e.g., pitting, stress, crevice, and fretting corrosion) [11].

**Figure 1:**
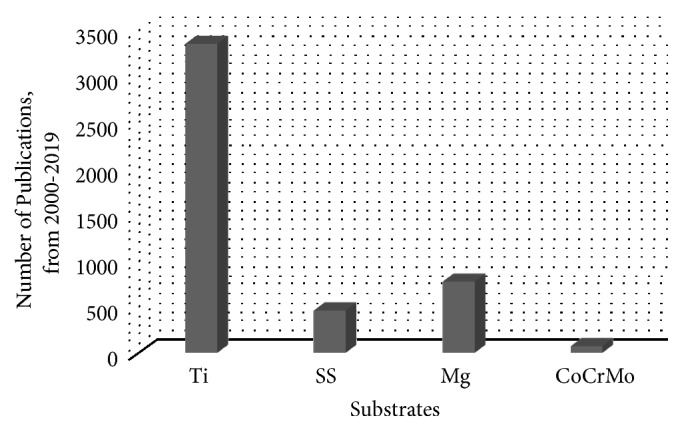
The approximate number of published articles of HAp deposition on different types of biomedical substrates from 2000 to 2019.

Titanium (Ti) and its alloys, stainless steel (SS), and magnesium (Mg) and its alloys are the most common substrates used for implant purposes [10, 12–17]. In addition, the use of CoCrMo alloys has also been studied as substrates [18].  Figure 1 shows the approximate number of published research papers from 2000 to 2019 for improving medical implants, including (but not limited to) corrosion studies, effect of cell growth in the presence of the implant, and various ways to improve adhesion of the HAp coating to the substrate.

Among these materials, titanium and its alloys are preferred because of a similar elastic modulus to that of bone and a naturally occurring oxide on the surface. Magnesium alloys and CoCrMo alloys have recently emerged for medical implant in vivo studies. Magnesium alloys are of interest due to the ability to safely degrade in vivo after the bone has healed. Surface activation of magnesium alloys is still desired because the implant needs to last long enough for bone regeneration. The use of CoCrMo alloys as an alternative to titanium alloys have been studied due to better mechanical properties especially higher surface strength which results in better corrosion resistance [19].

In this review, several different surface activation techniques will be comprehensively covered as a pretreatment for metallic substrates. These are pretreatments which involve etching in an acidic or alkaline media, soaking in H_2_O_2_, employing anodic oxidation, and sandblasting, as well as combining several of these techniques together with the addition of a heat-driven process to promote a surface transformation. Pretreating the substrate is done to help increase the interfacial bond strength between the metal substrate and HAp coating [9, 12, 20].

## 2. Surface Activation Techniques

### 2.1. Titanium Substrate

Titanium substrates and its alloys are extensively used among orthopedic and dental applications as load-bearing substrates due to their high mechanical properties and low elastic modulus. The elastic modulus of Ti (100 GPa) is more similar to bone (~30 GPa) than other materials, such as 316L stainless steel (210 GPa) and cobalt-chromium alloys (220-230 GPa) [9, 21, 22]. Ti metal also possesses good chemical stability and is biocompatible due to the passive oxide layer of titanium dioxide (TiO_2_) formed on its surface. The naturally formed titanium dioxide layer is a few nanometers thick (2-6 nm) [23] and is responsible for its chemical stability and biocompatibility. It is known that titanium will naturally form an oxide layer when expose to air and water. The function of the passive oxide layer is to eliminate releasing of metal ions into the human body to avoid harmful reactions and toxicity [24]. Much effort has been dedicated to increase the thickness of this oxide layer to improve its bone-bonding property and compensate for nonbioactive behavior [25]. The thickness of the oxide layer can be increased via chemical and thermal treatments to a few micrometers. Anatase and rutile phases are generally emphasized for crystalline TiO_2_ because they induce apatite-forming ability and stability more than other TiO_2_ phases. Various surface modifications have been investigated to encourage the TiO_2_ passive layer, leading to better adhesion and stronger bonds between the substrate and deposited hydroxyapatite film; these include alkaline, acidic, and H_2_O_2_ pretreatments.

#### 2.1.1. Alkaline Pretreatment

Alkaline pretreatments are often used for titanium substrates to create a hydrated titanium oxide gel layer. During the pretreatment process, hydroxide ions attack the titanium surface forming a sodium titanate (Na_2_Ti_5_O_11_) hydrogel layer [26]. The formation of the hydroxide groups on the surface of titanium during the alkaline pretreatment occurs as TiO_2_ first partially dissolves in alkaline solution; the reaction is presumed to continue with the hydration of Ti. The more hydroxide groups that react with the hydrate TiO_2_, the more negative the surface becomes. This leads to the formation of a sodium titanate hydrogel layer, this layer is unstable, and therefore, heat treatment is required to mechanically stabilize the layer. The mechanism describing the reaction occurring during the alkaline pretreatment process is shown below [26]:

(1) TiO2+NaOH→HTiO3−+Na+

(2) Ti+3OH−→TiOH3++4e−

(3) TiOH3++e−→TiO2∙H2O+0.5H2↑

(4) TiOH3++OH−↔TiOH4

(5) TiO2∙nH2O+OH−↔HTiO3−.nH2O

**Figure 2:**
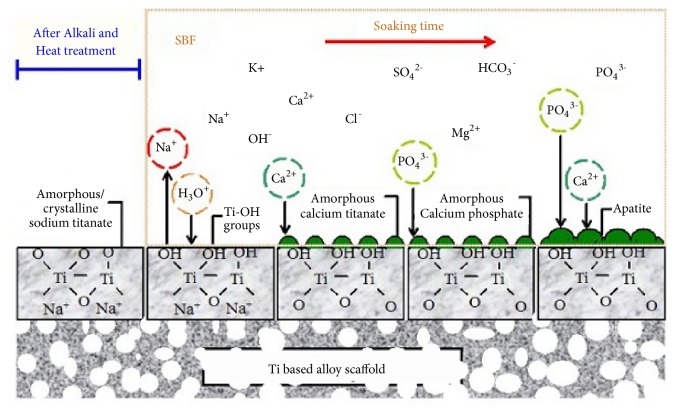
A schematic of apatite formation on the surface of alkali and heat-treated porous Ti based alloy scaffold soaking in SBF [26].

Figure 2 shows a schematic of the pretreatment process for the formation of apatite on the surface of titanium type alloy.

After the pretreatment process, the treated Ti substrate is immersed in a SBF solution. TiOH will form by releasing Na^+^ ions through ion exchange with H_3_O^+^ ions inducing apatite nucleation. The TiOH groups will create a localized negative charge and selectively bind with positively charged Ca^2+^ from the SBF solution, forming calcium titanate (CaTiO_3_) [27, 28]. The Ca^2+^ generates a positive charge on the surface, attracting PO_4_^ ^^3−^ ions to form apatite. The equilibrium in (6) illustrates the formation of HAp in SBF solution [26]:

(6) $$\begin{array}{c} 10{Ca}^{2+}+6{PO}_{4}^{3-}+{2OH}^{-}↔{Ca}_{10}{\left(\;{PO}_{4}\;\right)}_{6}{\left(\;OH\;\right)}_{2} \end{array}$$

Several studies have reported soaking the Ti substrate in 5 M NaOH for 24 hours at varying temperatures such as 60 or 80°C prior to electrodepositing the HAp coating. This results in a more bioactive calcium phosphate coating [27, 28]. After pretreating and electrodepositing a HAp coating on the Ti substrate, the substrate is ready for implantation. The bonding with the surrounding bones in the initial stages of implantation formed faster on the coating when using a NaOH treatment due to the increased surface area. Yanovska et al. [27] soaked the Ti alloys in 200 mL of 35% NaOH aqueous solution for 2 hours at 60°C and then for 48 hours at room temperature. This coating developed a dense HA composite layer in the form of an amorphous coating. The deposition of hydroxyapatite was achieved by a thermal substrate method (substrate temperature of 105°C, solution pH 6.5, 2 hr treatment) which developed a 1.04 mm thick and uniform coating on the surface.

After using an alkaline pretreatment, heat treatments can be applied afterwards to increase the crystallinity of the oxide layer. The oxide gel layer is formed by OH^−^ radicals attacking the Ti surface which transforms into crystalline titanate. Pan et al. pretreated Ti substrates in 5 M NaOH for 24 hours at 80°C followed by a rinse with distilled water and dried for 24 hours at 40°C [28]. The substrate was then heat treated for 1 hour at 600°C and cooled to room temperature. The alkali-heat treatment formed a porous and loose structure on the surface in addition to inducing heterogeneous apatite nucleation. The extended heat treatment ensures the oxide layer adheres to the metal substrate.

Alkaline pretreatment on the surface of titanium nanotubes was also studied by Parcharoen et al. [29]. First, anodization was done in an electrolyte solution containing 90 vol% glycerol and 10 vol% NH_4_F in water while applying a pulse voltage of either +20/-4 or +35/-4 V for 90 min to create a TiO_2_ layer. The anodized samples were then heated at 450°C for 30 minutes before alkaline pretreatment. The annealed, anodized titanium samples were then soaked in 1 M NaOH at 50°C for 2 minutes as a pretreatment process prior the deposition of HAp [29]. SEM scans of the Ti surface indicated that the nanotubes have a uniform shape when using +20/-4 V at both 5 and 25°C; however, the nanotubes formed a nonuniform shape when using +30/-4 V at both temperatures. The effects of alkaline treatment were also studied, on the surface of the untreated Ti substrate. An HAp coating was formed as an oriented rod-like structure with crystallite sizes around 100-300 nm. On the other hand, the coating appeared as unoriented rod-like structures on the surface of the pretreated Ti substrate with the crystallite sizes in the range of 100-200 nm. When comparing the difference between coatings on anodized Ti and conventional Ti, it was concluded that HAp coating appeared to be more adherent for the anodized Ti with OH- groups attaching better to the surface to form denser coatings. By forming the TiO_2_ nanotube geometry, the bonding strength between the coating and surface was significantly improved between the treated and untreated surfaces.

#### 2.1.2. Acidic Pretreatment

Acid treatments are implemented to increase the surface area and roughness of the substrate. The acid solution will initially remove corrosive free metals on the surface and then increase the thickness of the natural oxide layer. This will increase the contact and bonding between metal and HAp along with providing better crystallization of calcium phosphates. Hayakawa et al. etched Ti metal substrates in sulfuric acid (H_2_SO_4_) prior to a pulse current electrodeposition method to deposit HAp [25]. The substrates were soaked in different concentrations of sulfuric acid (25, 50, 75, and 97%) at 60°C for 30 min. Depending on the concentration of sulfuric acid, the XRD peak intensities of the Ti reflections would decrease or increase. For example, the intensity of the Ti (002) reflection decreased with increasing concentration of H_2_SO_4_. At a high concentration of 97% H_2_SO_4_, the surface was similar to the untreated surface due to the inactive nature of the Ti metal towards oxidizing acids. Adhesion was greatly improved when etched in 50 and 75% H_2_SO_4_. As a posttreatment, the HAp-coated substrates were heated at 600°C for 60 min. The heat treatment enhanced the adhesion even further by decreasing the HAp crystallite size.

Hydrofluoric acid (HF) is a commonly used acid for treatment of medical implants, to help improve the bond response and better implant attachment [30]. Soaking in 1 and 40% HF for 1 min at room temperature reduces the hydrocarbon surface content, which increased the surface energy and potential of bioacceptability for the titanium substrate [30]. Pure titanium commercial samples were annealed at 950°C for 1 hr before immersion into acidic solution. XPS was used to analyze and study the characteristics of the titanium surface before and after acidic treatment. Although HF pretreatment induced faster HAp formation, HAp coated on an untreated substrate exhibited a higher crystallinity than the treated substrate. The faster formation of HAp was not favorable, since the pretreated substrate was less crystalline than the untreated substrate. However, after implementing HF pretreatment, the HF treated samples reduced surface contaminations and increased the TiO_2_ layer thickness. Yanovska et al. studied the effect of pretreatment on the surface of titanium using 10% aqueous solutions of HF and compared to pretreating methods using H_2_O_2_ or NaOH [27]. The researcher found that etching the surface using HF created a negative charge surface that increased the rate of Ca^+2^ ions attaching to the substrate. HF pretreatment resulted in a more crystalline structure with needle-like crystals of HAp on the surface compared to the other pretreatment methods. Overall, Yanovska et al. [27] concluded that the high crystalline surface lends itself towards better surface modification.

The treatment of pure titanium using 5 wt% oxalic acid at 100°C followed by the thermal oxidation at 450°C for 2, 4, and 6 hr was studied by Wang et al. [23]. After etching with acid solution, the surface contained a thin layer of titanium oxide (3-7 nm as TiO_2_). However, after the thermal oxidation process, the thickness of the oxide layer increased dramatically, for samples heated for 2-4 hr (30-50 nm) and for samples heated for 6 hr (100-150 nm) [23]. Samples that were kept for 6 hr in the oven were found to have the highest W_R_ (the relative weight percentage of rutile), lower contact angle, and better osteogenic capacity in both* vitro* and* vivo. *

Pretreatments in phosphoric acid have also been shown to be effective. Immersing Ti substrates in 1-2% (w/w) H_3_PO_4_ solutions at 180°C for 2 hours in a Teflon-lined reactor, followed by a subsequent heat treatment at 400°C for 12 hours have significantly increased wettability, osteoblast cell response, and bone-implant contact and exhibited a microrough surface structure [24]. Phosphorus ions incorporated into the Ti surface was characterized as a crystalline titanium oxide phosphate hydrate film on the surface, Ti_2_O(PO_4_)_2_(H_2_O)_2_.

#### 2.1.3. H_2_O_2_ Pretreatment

A H_2_O_2_ pretreatment is an effective way to increase the bioactive properties of calcium phosphate coatings because it increases the surface area of the substrate, induces a bone-like apatite layer in a shorter period of time (during electrodeposition and/or SBF immersion), and provides more favorable sites for calcium phosphate nucleation. H_2_O_2_ oxidizes the titanium to form an anatase-type TiO_2_ film with low crystallinity (TiO_2_ gel) on the surface, precipitating as titanium oxide or titanium hydroxide. The oxidation process is shown in (7) [27, 31]

(7) $$\begin{array}{c} Ti+3H_{2}O_{2}\to {\left[\;Ti{\left(\;OH\;\right)}_{3}O_{2}\;\right]}^{-}+H_{2}O+H^{+} \end{array}$$

The formation of TiOH groups on the surface is an advantageous precursor to the formation of apatite, as shown for Figure 2. The formation pathway for HAp on the titanium-treated surface in SBF solution is shown in (8) and (9) [27].

(8) 2H++TiOH3O2−+Ca2++2OH−→Ca2++TiOH3O2−+2H2O

(9) 5Ca2++3H2PO4−+7OH−→Ca5PO43OH+6H2O

There are several variations of H_2_O_2_ treatment; a few are shown in Table 1. Ueda et al. implemented a chemical-hydrothermal treatment by using a combination of hydrogen peroxide/nitric acid and UV irradiation [31]. Compared to the other methods this one was more tedious, since the disks submerged in the baths were put in a Teflon-lined autoclave at 453 K for 12 hours before starting the UV irradiation process. However, the effect of the UV irradiation on the surface of the substrate provided uniform 40 nm cubic crystals. The formation of HAp on TiO_2_ in SBF contained a large number of spherical clusters and a thin homogenous film was attained.

#### 2.1.4. Anodic Pretreatment

The characteristic properties of the oxide layer can be tailored by altering the parameters of the anodization process (oxidation) in addition to incorporating valuable chemical species from the electrolyte solution. Electrode reactions in collaboration with field-driven ion diffusion during the process of anodization form an oxide layer on the anode when passing a constant voltage between the anode and cathode [33]. Using different electrolyte solutions, electrolyte pH, anodization time, and applied potential will affect the crystallinity and morphology of the oxide film. Titanium oxide naturally grown has a thickness of 2-6 nm; in order to increase the thickness of this oxide layer, anodic oxidation is a good choice due to its low costs, simplicity of the experiment, and control of the coating's thickness [34]. For titanium, the electrolyte may consist of a variety of acids, neutral salts, and alkaline solutions; but, acidic electrolytes are generally favored due to higher affinity for oxide formation compared to other electrolytes [35]. This preferred pretreatment process can be conducted on irregular substrates and allows easy and simple control of crystal growth.

The addition of fluoride ions (~0.05-0.5 M F^−^) in the electrolyte solution is a strategic additive for forming self-ordering TiO_2_ nanoporous structures via anodic oxidation. Fluoride ions containing electrolytes have two important roles: (1) react with Ti^4+^ ions which are dissolved at the oxide-electrolyte interface to form a soluble [TiF_6_]^2-^ complex and (2) chemically dissolve TiO_2_ to form a [TiF_6_]^2-^ complex [9, 33, 36]. Accomplishing these two roles leads to the formation of the [TiF_6_]^2-^ complex, as shown in (10)-(12) [10, 37].

(10) Ti+2H2O→TiO2+4H++4e−

(11) Ti4++6F−→TiF62−

(12) TiO2+6F−+4H+→TiF62−+2H2O

Through these reactions and the effect of F^−^ etching, the assemblies of self-ordering TiO_2_ nanoporous structures are established. Yan et al. obtained uniform nanotubes by anodizing in 5 wt% HF electrolyte for 60 min at room temperature using a potential of 20 V via a direct current power source (Ti sheet as the positive terminal and platinum foil as the negative terminal) [37]. This process created a TiO_2_ nanotube layer with diameters of 100 nm, increasing the formation of apatite (via electrodeposition of HAp) and enhancing the bond strength by more than 15 MPa through the anchoring effect. Using a pulse anodization technique, Parcharoen et al. electrochemically anodized TiO_2_ nanotube layers on a titanium substrate using ammonium fluoride (NH_4_F) electrolyte containing viscous modifiers, such as glycerol or polyethylene glycol [10]. To further homogenize the nanotube arrays, an alkaline treatment of 1 M NaOH at 50°C for 2 min was used on the anodized titanium, forming sodium titanate (Na_2_Ti_3_O_7_). The anodization time affected the length and wall thickness of the TiO_2_ nanotubes. When the anodization time was too short, the TiO_2_ nanotube arrays became irregular due to an initial higher growth rate at the beginning. In contrast, a longer anodization time leads to the individual pores interfering with each other and a decrease in adhesion. The longer analysis time causes the TiO_2_ layer to change structure, altering the mechanical interlocking between the HAp coating and nanotube arrays. It was concluded that a viscous electrolyte solution consisting of 10% NH_4_F in water with 90% glycerol (viscosity of 300 cP) made the most improvement and obtained the highest uniformity when combined with a pulse anodization time of 1.5 hours (560 nm length, 10 nm wall thickness). This is because the NH_4_^ ^^+^ ions bind with TiO_2_ forming TiO_2_(NH_4_^ ^^+^), protecting the nanotube walls against chemical etching by fluoride ions [10]. The addition of modifiers assists in the regulation of local concentration and pH fluctuations, resulting in smooth and uniform TiO_2_ nanotube arrays. The improved adhesion enhanced bone formation through increased surface area and created a physical locking between the HAp and anodized titanium substrate.

**Figure 3:**
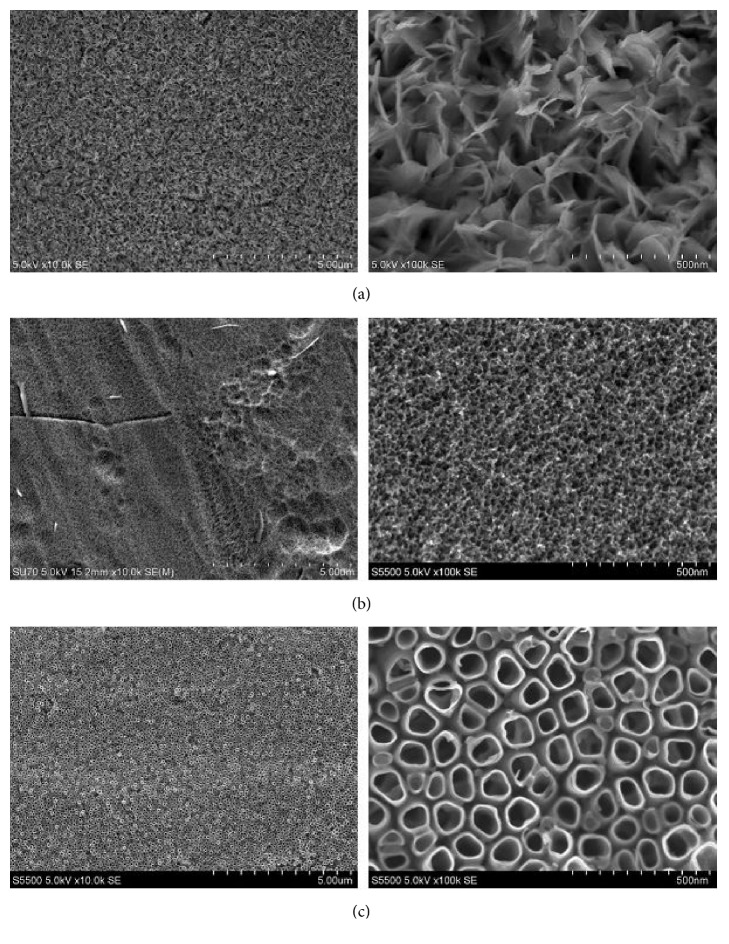
SEM images of chemically treated Ti in (a) NaOH, (b) H_3_PO_4_ + H_2_O_2_ solution, and (c) electrochemically treated in NH_4_F + glycerol + water electrolyte (20V for 2 h) [38].

Another study deposited a calcium phosphate coating onto titanium substrates that were treated utilizing either chemical or electrochemical method [38]. Titanium substrates were treated using a chemical pretreatment by either soaking in a 3 M NaOH aqueous solution for 24 hr at constant temperature (70°C), or soaking in H_3_PO_4_ + H_2_O_2_ solution for 24 hr at room temperature. The electrochemical pretreatment of titanium was performed to create titanium oxide nanotube layers utilizing anodic oxidation in the electrolyte that consists of NH_4_F (0.86 wt%) + DI water (47.14 wt%) + glycerol (52 wt%) at room temperature. The applied voltages were maintained in the range of 10-25 V. The samples were sintered at 600°C for 1 or 2 hr. The morphology of the titanium substrates after chemical and electrochemical pretreatments was analyzed using SEM (Figure 3) [38]. After immersion in 3 M NaOH, the titanium surface developed a layer of sharp-edged pores in different shapes (Figure 3(a)). However, after pretreatment with H_3_PO_4_ + H_2_O_2_ solution, the titanium surface appeared more sponge-like and uniform compared to the previous treatment (Figure 3(b)). Lastly, electrochemical pretreatment resulted in a very compact surface with the formation of TiO_2_ nanotubes (Figure 3(c)); these nanotubes were evenly separated from each other on the substrate. The diameter of the nanotubes increased as the applied voltages increased (40 nm for 10 V to 110 nm for 25 V).

Anodic oxidation of a titanium surface was also studied using sulfuric acid (H_2_SO_4_) by Vera et al., the electrolyte concentration varied from 0.1 to 4 M, and the applied voltages varied from 20 to 70 V [34]. After the oxidation process, samples were rinsed with DI water and dried under hot air. A set of samples that were pretreated at different electrolyte concentrations (0.1-4 M) were analyzed at different voltages (20 – 70 V); as the electrolyte concentration increased, the color of the sample started changing. At 20 V, the samples went from dark blue/orange to yellow/green for different concentrations; at 40 V, the samples went from light orange to yellow; at 60 V, the samples went from dark orange to red; at 70 V, the samples went from yellow to purple and pink. The color changes were due to the higher concentration and conductivity of the electrolyte affecting the growth rate or changing the orientation of the phases on the substrate [34]. However, the morphology of the surface significantly changed from amorphous to crystalline, with an increase in applied voltage but not with an increase in acid concentration. In conclusion, the best coating was formed in 4 M H_2_SO_4_ using 60 V as the applied potential; 70 V could also be used with lower concentration of the electrolyte.

In the last decade, there have been a few reports of anodizing in phosphoric acid solutions. Anodizing in phosphoric acid based solutions has shown stimulation in cell proliferation on the oxide layer due to the incorporation of phosphorus into the layer. Depending on the applied voltage, the oxide layer characteristics are drastically different. Low voltages induce thin, compact, and amorphous oxide layers while high voltages (past the breakdown potential) exhibit thick, porous, and crystalline oxide layers. A study carried out by Chen et al. evaluated the effect of pure titanium substrates anodized in phosphoric acid at different applied voltages [35]. The process was conducted at room temperature in a 1 M phosphoric acid solution using a DC power supply. Each pure titanium plate was anodized for 2 min at 100, 200, and 300 V. All three applied voltages exhibited significantly different characteristics.

At 100 V (below the breakdown potential), a dense and uniform oxide layer formed which was also composed of grainy particulates in the nanometer range. At potentials past the breakdown potential, 200 V and 300 V, a porous microstructure with craters and pores on the surface was obtained (no observed nanostructures). The craters and pores created at 300 V were much larger than the pores created at 200 V. The breakdown potential is influenced by the concentration of the electrolyte solution; the breakdown potential decreases with increasing electrolyte concentration. When the breakdown potential is reached, discharges will initiate at the weaker regions of the oxide layer forming pores. Poor crystallinity with no indications of TiO_2_ was observed for 100 V and 200 V; in contrast, anatase-TiO_2_ was apparent when the voltage was increased to 300 V. However, the incorporation of phosphorus in the oxide layer may suppress the crystallization of the anodic oxide layer to some extent. Although high crystallinity was observed at 300 V, the highest number of attached cells was achieved on the oxide layer created at 100 V due to the biomimetic nanostructured surface topography. Cell adhesion was most favored for this morphology by one order of magnitude, promoting cell proliferation.

The morphology will also drastically differ when different electrolyte solutions are utilized. By combining different amounts of phosphoric acid and hydrofluoric acid, PO_4_^ ^^3−^ and F^−^ ions become competitive when intercalating into the oxide layer. Kim et al. explored this phenomenon by anodizing titanium foils (99.6%) in various solutions; results listed in Table 2 [39].

When using only HF as an electrolyte, the TiO_2_ layer showed dot-like structures, indicating the formed oxide layer was rapidly dissolved in solution. With the addition of phosphoric acid, nanopowder consisting of granules (<100 nm) was produced on the dot-like surface. The H_3_PO_4_ delays oxide dissolution. As the concentration of H_3_PO_4_ increased, the size of the granules reduced, leading to the formation of nanopowder and nanotubes (~200 nm in diameter). Short length nanotubes (100 nm) could be formed in a mixture of 1 M H_3_PO_4_ and 1 wt% HF at lower potentials, such as 20 V.

Many other studies have been done comparing electrolyte solutions for the film growth of TiO_2_ on titanium [40, 41]. Liu et al. studied anodization of titanium in sulfuric and phosphoric acids [41]. Stable barrier anodic films could be formed by applying 10 to 60 V (vs SCE) in either 1 M H_2_SO_4_ or 1 M H_3_PO_4_. The oxide thickness increased with increasing applied voltage. The higher the applied voltage, the larger the nanotubes that were formed (from 25-45 nm to 50-100 nm).

**Figure 4:**
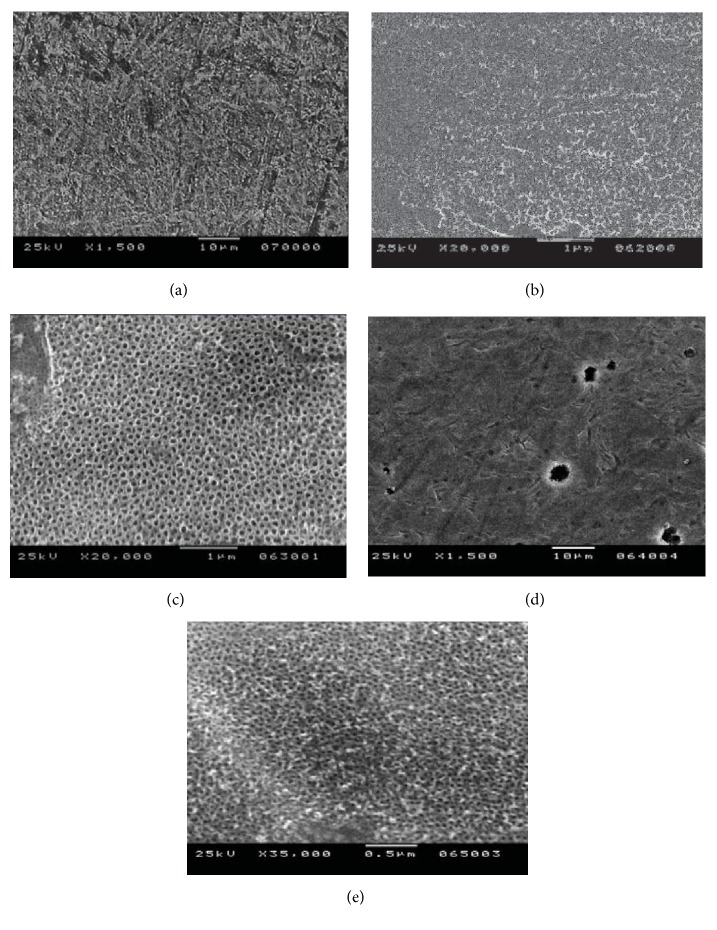
SEM images of titanium oxides that are anodically prepared under different anodizing conditions: (a) chemical etch in 0.5 wt% HF for 30s, (b) aqueous 0.3 wt% HF + 1 M H_3_PO_4_ at 20 V, (c) aqueous 0.5 wt% HF + 1 M H_3_PO_4_ at 20 V, (d) aqueous 0.5 wt% HF + 1 M H_3_PO_4_ at 10 V, and (e) aqueous 0.5 wt% HF + 1 M H_3_PO_4_ at 150 V [42].

The effect of the various electrolyte solutions on the morphology of titanium can be seen in Figure 4 [42]. For this study HF and H_3_PO_4_ mixtures were used as electrolyte during anodic oxidation of titanium.

As in other studies, the anodization potential had a strong effect on the morphology of the surface. Anodizing the Ti alloy in 0.5 wt% HF + 1 M H_3_PO_4_ at 20 V produced ordered nanotubes with 80 nm diameter (Figure 4(c)). The anodizing potential also affected the nanotube diameter. 200-250 nm oxide layer thickness was produced for processing times of ~2 hr.

#### 2.1.5. Sandblasting

**Figure 5:**
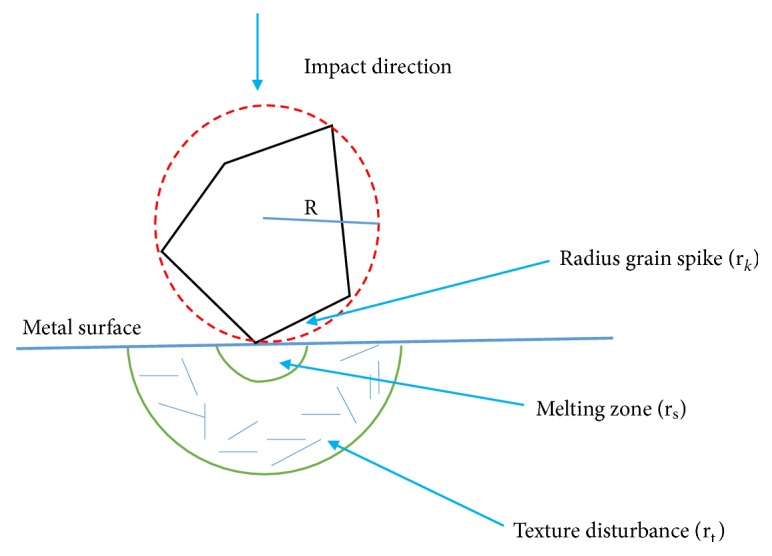
Variations of a metal surface at the impact point of a grain during sandblasting process. R: radius grain; r_k_: radius grain spike; r_s_: radius melting zone; r_t_: radius texture disturbance [43].

Sandblasting is an abrasive technique used to eject a high pressure stream of material against a surface for modification such as cleaning, roughening, and activating metal surfaces [43]. Once the sandblasted material has impinged on the metal surface, the impact causes a momentum and kinetic energy transfer, creating a large area of lattice defects. This is initiated by the crystal lattice absorbing the kinetic energy executing surface melting on a microscopic range. This process is shown in Figure 5.

Corundum (Al_2_O_3_) is commonly used as the carrier material for sandblasting applications of materials used in dentistry and orthopedics; Al_2_O_3_ has been chosen due to its hardness, particle shape, and low cost. This is a nonsolution process that can also be used to prepare metallic substrates. Gbureck et al. coated a corundum core with TiO_2_ and hydroxyapatite porous shells, thus using the alumina core as a carrier material, to sandblast layers onto a titanium surface [43]. A blasting pressure of 0.4 MPa for 20 s/cm^2^ was used. This method reduced contamination with corundum and reinforced the native oxide layer of titanium. Alumina particles were also used for the sandblasting process on the surface of Ti-6Al-4V alloy by Balza et al. [44]; the samples were sandblasting at 0.3 MPa pressure, 90° angle, using 420-600 *μ*m alumina particles; each sample was polishing between 2 and 10 seconds. The sample surface was characterized using SEM before and after sandblasting. SEM images showed that the roughness of the titanium alloy surface increased after the blasting treatment, the optimum roughness was 3.4 *μ*m at 7 s, but the roughness of the surface went down to 3.1 *μ*m at 10 s, which indicated that the surface tended to become smother as the samples were treated longer than 10 second. Sandblasting with corundum is not limited to titanium, but applicable to other materials like stainless steel and CoCr-alloys.

#### 2.1.6. Combining Techniques

Techniques such as sandblasting, acid etching, and anodic oxidation can be combined together to modify the surface of a titanium substrate and create a nanoporous surface structure. For example, hydroxyapatite was electrodeposited onto a titanium substrate and the bonding strength, coating adherence and morphology was studied by comparing the pretreatment method for the titanium before deposition [45]. Ti plates (10 × 10 × 1 mm) were polished using 200, 400, 600, and 1000 grit sandpaper, followed with sandblasted at 0.3 MPa for 30 s using quartz sand. After the treatment, sandblasted (SB) samples were ultra-sonicated in water to clean off the extra residual. These samples were next immersed in 49 wt% sulfuric acid at 60°C for 1 hr; the samples that were both sandblasted and treated with acid were labelled Ti (SBA) samples. Lastly, these Ti (SBA) samples were anodized in a glycerin-water electrolyte (v:v 1:1) with 10 g/L NH_4_F at 20 V for 1 hr followed by heating at 450°C for another hour. Nanobrushite coating was electrochemically deposited on the substrates from an electrolyte solution containing 10 g/L Ca(NO_3_) and 4 g/L (NH_4_)_2_HPO_4_ at 3 V for 1 hr. Finally, the samples were cleaned with acetone, ethanol, DI water and dried at 40°C. After the surface treatment process, all samples were immersed in SBF solution for 1, 3, 7, and 14 days at 37°C, SBF solution was refreshed every other day. XRD was used to analyze the Ti substrate before and after the deposition and, as a result, the intensity of the brushite peaks from the anodized Ti (SBA) sample had the highest intensities with preferred orientation of the (020) plane. Also, brushite on the surface of anodized Ti (SBA) sample appeared to be the most homogeneous structure with a thickness of about 80 nm [45].

### 2.2. Stainless Steel Substrate

**Figure 6:**
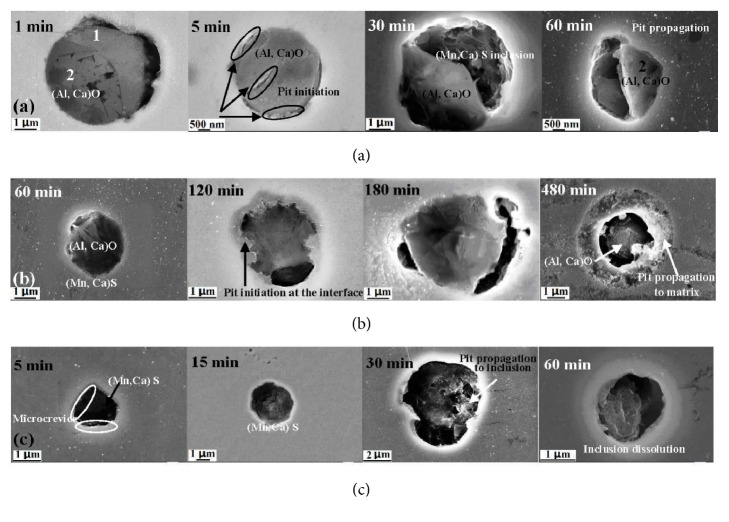
SEM images of three types of inclusions after initiation and propagation of pitting corrosion in X70 steel: (a) Type A (particles of (Al, Ca)O and (Mn,Ca)S); (b) Type B ((Al,Ca)O), and (c) Type C ((Mn, Ca)S). Steel was immersed in 0.1 mol/L NaCl and 0.5 mol/L NaHCO_3_ solutions at 25°C for times indicated in figure [50].

Austenitic grade AISI 316L stainless steel is also widely used as a metal for medical and dental applications [46, 47]. Stainless steel (SS) contains different ratios of chromium (Cr) and other metals such as manganese, nickel, iron, and molybdenum. SS can eventually rust, creating a corrosive iron oxide layer, when exposed to air and/or water. The chromium within the SS creates a protective oxide layer on the surface; thus, the higher the chromium content, the lower the corrosion rate. At a minimum of 10.5% Cr content, SS exhibits a natural Cr_2_O_3_ film (1-10 nm thickness) when exposed to oxygen but it is not as strong as when passivated [13]. When the metals on the surface are not sufficiently alloyed with chromium, rust is formed. Passivation of SS occurs by first removing any free iron or manganese sulfide (MnS) inclusions on the surface, usually by an acid, to eliminate contribution to corrosion defects. MnS inclusions are defect points for pitting corrosion to occur on the SS surface, initiating discontinuities of the passive film (see Figure 6 for examples of inclusions) [48–50].

Once treated, the chromium in the SS will be oxidized to chromium oxide (Cr_2_O_3_) forming a protective layer. Chromium is known as a passive promoter due to the combination of strong chromium-oxygen bonding as opposed to low metal-metal bond strength, favoring the stability of the passive film and rapid nucleation and growth of the oxide [48, 49]. Passive promoters are not limited to just chromium, but also include other elements such as titanium and aluminum. In vivo corrosion of SS occurs from release of metallic ions such as Ni^2+^, Cr^3+^, and Cr^6+^ and affects proliferation and differentiation of cells in addition to being powerful allergens and carcinogenic [49, 51]. The following pretreatments are emphasized in order to reduce corrosion and wear rates in addition to increasing the lifetime of the coating and bond strength with HAp.

#### 2.2.1. Alkaline Pretreatment

Alkaline pretreatments create a metal-OH layer on the surface of the substrate, much like the treated-titanium substrates. Once immersed in an alkaline solution, the substrate forms a metal oxide layer which dissolves to form metal hydroxide creating a hydrous gel layer. The alkaline treated substrate can then be exposed to a SBF solution in which Ca^2+^ and Mg^2+^ will adsorb via ion exchange, inducing calcium phosphate nucleation [52]. The metal-OH layer is the key to calcium phosphate nucleation, for metallic substrates.

A thermal oxidation technique has been used to increase the thickness of the chromium oxide layer. This has been accomplished by placing the substrate in a resistance furnace at temperatures ranging from 400–1200°C [51]. Corrosion resistance of the surface occurs with passive film formation. Lin et al. alkali-treated 316L SS substrates in 10 M NaOH at 60°C for 24 hours and after rinsing and drying at 40°C for 24 hours, the samples were subsequently heated to 500-800°C (5°C/min) in a furnace for one hour [52]. Heating the alkali-treated substrate at different temperatures showed an interesting trend. The hydrate phase transforms into sodium chromium oxide (Na_4_CrO_4_) at 600°C, but phases out once the temperatures was increased to 700-800°C where iron oxide (Fe_2_O_3_) and iron chromium oxide (FeCr_2_O_4_) start appearing. The appearance of iron in the passivation layer causes instability in the film, further leading to the interface layer peeling off. Subsequent heat treatment at 600°C was most optimal, where the assumed reaction is denoted in (13) [52].

(13) $$\begin{array}{c} 8Na\left(\;OH\;\right)+{Cr}_{2}O_{3}\to 2{Na}_{4}{CrO}_{4}+3H_{2}O+H_{2} \end{array}$$

Heat-treating above 600°C induces a weak passive layer derived from the loose structure of iron oxide and iron chromium oxide, decreasing the bonding strength from the substrate to the film. The chromium oxide layer is the initial protective coating on the 316L SS surface with Na_4_CrO_4_ forming on top after alkali-treatment. The Na_4_CrO_4_ layer is the interlayer “link” that strongly bonds with HAp and chromium oxide.

#### 2.2.2. Acidic Pretreatment

Acidic pretreatments are very efficient and effective. The acid removes MnS inclusions in addition to creating a strong passive layer on the substrate by oxidizing the chromium content and encouraging noble element enrichment [53]. S. Kanaan et al. explored the effects of acid pretreatment on 316L SS with sulfuric acid [13]. For sulfuric acid treatments, 316L SS substrates were completely submerged in 5 to 20% H_2_SO_4_ for 1 hour at room temperature; subsequently rinsed with distilled water; and dried at 50°C. The passive layer of this acid treatment was extensively explored through electrochemical studies such as cyclic polarization and impedance spectroscopy. Energy dispersive x-ray analysis (EDAX) and inductively coupled plasma atomic emission spectroscopy (ICP-AES) were used to observe the leeching of metals from the substrate. Among the various H_2_SO_4_ treatments used, 15% concentration was optimal. The breakdown potential of the cyclic polarization results indicated a maximum E_b_ value of +680 mV, almost double the value of pristine 316L SS (+320 mV), indicating a shift towards a nobler direction. Impedance results indicated a max polarization resistance (R_p_) value of 126.2 Ω and electrical impedance (|Z|) value of 2.09 in 15% H_2_SO_4_ as opposed to untreated 316L SS (R_p_ value of 43.72 Ω, |Z| value of 1.61). These results are believed to be due to the presence of chromium oxide and Mo enrichment. Substrates will form strong passive layers when noble alloying elements are present. Studies have proven that enhanced passivating behavior is derived in stainless steel when Mo, a noble alloying element, is present and exposed to H_2_SO_4_ [53]. To prove this, EDAX and ICP-AES were utilized to show the concentration of different metals on the surface after immersion in various H_2_SO_4_ concentrations. At 15% H_2_SO_4_, higher amounts of Cr and Mo were present and lower amount of Fe as compared to untreated 316L SS. The iron content increased and the Cr and Mo content decreased when the 316L SS substrate was submerged in 10 and 20% H_2_SO_4_. These studies indicate the strong beneficial influences on pitting resistance and wear rate of stainless steel when Mo and Cr are integrated.

Nitric acid and phosphoric acid pretreatments have similar effects on 316L SS surfaces, much like sulfuric acid [49, 54]. Noh et al. studied nitric acid passivation effects on 316 SS by immersing the substrates in nitric acid up to 50% for 1 hour at room temperature. Results indicated an effective increase in chromium enrichment of the passive film and MnS inclusions were removed from the alloy surface when treated in 20-25 wt% nitric acid [49].

#### 2.2.3. Electron Beam Surface Pretreatment

Bombarding the substrate with highly energetic particles is another type of surface pretreatment that can be used to enhance corrosion resistance and bonding of HAp in steels. High energy, low current DC electron beam surface treatment was applied to surgical grade stainless steel by Gopi et al. [55]. In this process, crater eruptions are created at MnS inclusions, producing a surface purification effect and nucleation sites. The SS surface becomes completely melted and solidified from the electron beam irradiation creating strong interfacial bonding between the melted region and substrate, preventing surface oxidation, and eliminating the formation of pores and cracks derived from the heating and cooling effect. The 316 SS specimen was surface treated with an electron beam of energy 500 keV, beam current 1.5 mA, using a 700 keV DC accelerator, passing through the beam at 20 m/min (two passes, 30 s separation). When HAp was electrodeposited on the treated substrate, the morphology of the HAp coated SS-treated substrate exhibited microstructured flowers (nonuniform nanorods/nanoflakes) with a thickness of 90-150 nm, possibly due to the erupted sites on the surface. According to the potentiodynamic cyclic polarization studies, the treated-316L SS manifested a high resistance in Ringer's solution. Compared to the untreated HAp-coated substrate, the treated substrate exhibited a maximum shift in the noble direction with values of 520 and 172 mV at the breakdown potential (E_b_) and repassivation potential (E_p_), respectively.

### 2.3. Magnesium Alloy Substrate

**Figure 7:**
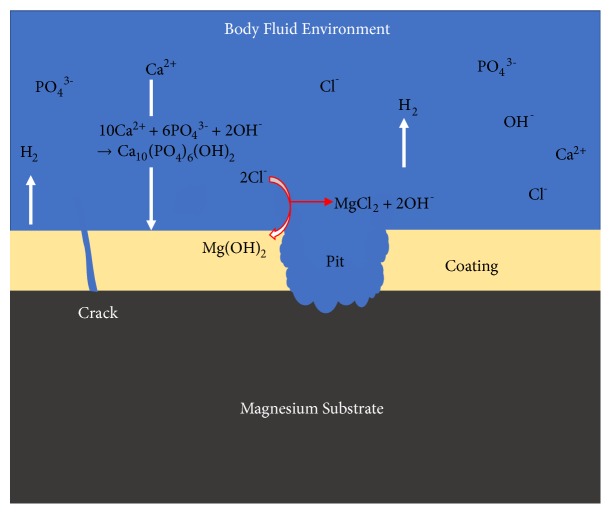
Schematic diagram illustrating the corrosion failure and species present for surface modified magnesium and its alloys.

Magnesium alloys have become popular mainly because these alloys exhibit good biodegradable characteristics [56]. Although Mg alloys have advantages compared to inert metallic biomaterials, e.g., good mechanical properties, biocompatibility, and strength-to-weight ratio, the alloys also have poor corrosion resistant properties in chloride solutions [57]. Magnesium alloys are recognized as alternatives to stainless steel and aluminum alloys due to its lower weight and high strength-to-weight ratio. Although magnesium implants tend to release an acceptable amount of metallic ions into human body, these ions could be absorbed around the tissues and eventually released via the kidneys. On the other hand, magnesium also degrades after implantation which could eventually be harmful for the body or delay healing times [58, 59]. Pitting of the magnesium is initiated when the chloride ion concentration reaches 0.002-0.02 M NaCl; chloride ions absorb onto the oxide film transforming Mg(OH)_2_ to soluble MgCl_2_ [60]. The species present during pitting corrosion on the surface of magnesium is described in Figure 7.

The elastic modulus of pure magnesium (45 GPa) is actually closer to that of bone than titanium; however, the poor corrosion resistance leads to implant degradation before the healing process is over. This creates hydrogen evolution, which delays the healing process or can cause death and alkaline poisoning [60, 61]. The average rate of hydrogen evolution for specific magnesium alloys has been examined by Song et al.; the volume of evolved hydrogen (ml/cm^2^) was observed for 30 days and is shown in Table 3 [60]. Commercial purity magnesium (CP-Mg) showed an average rate of hydrogen evolution of 26 mL/cm^2^/day, which corresponds to a measured weight loss of 19-44 mg/cm^2^/day. Magnesium alloys containing aluminum AZ91D (8-10% Al) and containing zinc ZE41 (3-5% Zn) had an average rate of hydrogen evolution of 0.068 and 1.502 mL/cm^2^/day, respectively. These values verify that alloying can retard the biodegradation process for Mg.

Mg and its alloys immersed in neutral SBF solution will raise the pH of the solution to ~11 and the pH at the surface will always be above 10 [62]. The local alkalization can affect the physiological pH reaction balances around the Mg implant and result in an alkaline poisoning effect if the in vivo pH value exceeds 7.8. Slowing down the biodegradation rate of Mg alloys will also slow down the generation of Mg^2+^ ions, H_2_ evolution, and OH^−^ ions so that the human body can gradually adjust. The electrochemical degradation of Mg in aqueous solutions is denoted in (14) and (15) [59].

(14) Mg+2H2O→Mg2++2OH−+H2

(15) Mg2++2OH−→MgOH2

Thus, research on magnesium alloys for implant applications is focused on decreasing the degradation rate. The larger the difference in elastic modulus between the implant and the host hard tissue is, the more stress shielding effects take place in the bone tissue [60]. Compared to titanium, the stress shielding effects could be greatly reduced if magnesium became the alternative. A natural oxide layer can form on the magnesium surface but exhibits a loose structure and cannot offer an effective resistance to corrosion. Therefore, several surface modifications such as anodizing and etching in alkaline or acidic solutions have been applied to modify the surface reactivity of the magnesium alloy substrate [63, 64]. Surface modification provides a foundation for HAp to adhere to, providing a barrier between the substrate and the aggressive environment, allowing the substrate to gradually release magnesium ions into the human body at an optimal degradation rate. The types of surface modifications that can be accomplished for Mg alloys are discussed in the next sections.

#### 2.3.1. Alkaline Pretreatment

Alkaline pretreatment for Mg has several advantages. The conversion coating caused by alkaline pretreatment increases particle boundaries and surface roughness and may also aid towards protein interactions, cell adhesion, and tissue integration [63]. Grubač et al. used a one-step alkaline pretreatment prior to electrodeposition of HAp. A degreased magnesium alloy (AZ91D, wt.%: Al 8.6, Mn 0.19, Zn 0.51, Si 0.05, Cu 0.025, Fe 0.004, and balance Mg) substrate was immersed in 1.0 M NaOH solution at 80°C for 1 hour and then rinsed with distilled water [63]. After electrodeposition of calcium phosphate, an immersion test was repeated as a post treatment for 2 hours. The end product of HAp exhibited needle-like dendrite structure and a calcium deficient coating. Deposits of calcium deficient HAp possess good bioresorption.

Alkaline treatments have also been used in combination with other treatments. The combination of alkali and heat treatment has shown to keep the pH lower during the degradation of pure magnesium (99.99%). This process was accomplished by soaking pure magnesium in a super saturated solution of NaHCO_3_-MgCO_3_ for 24 hours at a starting pH of 9.3 followed by a heat treatment at 773 K for 10 hours [65]. The mass of the alkali-heat-treated pure Mg substrates remained constant for 14 days and the surface morphology maintained a smooth surface for 7 days, indicating good corrosion resistance in SBF. The pH of the SBF solution was also monitored during immersion of the treated and untreated Mg substrates. The untreated samples raised the bulk pH above 10.5 just after 6 days (pH 9 at day 2); in contrast, the alkali-heat-treated samples reached pH 9.5 after 5 days (pH 8.25 at day 2) but remained constant up to 14 days. The two-step treatment proved effective due to the slower rate of pH increase. Mg-Ca alloy samples have also been investigated with other types of alkali-heat-treatments in Na_2_HPO_4_, Na_2_CO_3_, and NaHCO_3_, all followed by a 12 hour heat treatment at 773 K in air [64]. Although all showed improvement compared to the pristine substrate, NaHCO_3_ heat-treated Mg-Ca alloy showed the most uniform, dense, and thick surface, successfully slowing the rate of corrosion and providing good protection for the substrate.

Gray-Munro et al. used a four step pretreatment process on magnesium aluminum zinc foil (96% Mg:3% Al:1% Zn by weight) to induce calcium phosphate deposition from aqueous solution by increasing the number of hydroxyl groups on the surface which had already been proven to work on other materials like titanium and stainless steel [14]. The four-step treatment process included (1) sonication in trichloroethylene (30 minutes, room temperature) and then rinsing with distilled (DI) water, (2) sonication in Na_2_CO_3_ (25 g/L) (30 min, 50°C) and then rinsing with DI water, (3) alkaline aging (200 g/L NaOH, 24 hours, room temperature) and then rinsing with DI water, and (4) heat treatment (140°C, 24 hours). Although XPS studies showed the presence of Mg(OH)_2_ which could lead to promotion of hydroxyl groups on the surface from pretreating in NaOH solution, the characterization of the HAp deposited on pretreated Mg alloy resulted in a poorly crystalline calcium magnesium HAp material. This was due to the anodic dissolution of the Mg alloy substrate during the early stages of the nucleation and deposition of the calcium phosphate coating [14].

#### 2.3.2. Acidic Pretreatment

Mg alloy surfaces can also be modified with acid pretreatment. Etching in F^−^ containing solutions forms a protective conversion coating on the substrate. Fluoride ions have a desired ability to form water soluble metal-fluoride complexes, developing self-ordered nanoporous and nanotublar oxide layers [36]. Mg-Zn-Ca alloys have shown improved corrosion resistance and biocompatibility when activated with 40% HF for 10 min before using a pulse electrodeposition method [66]. Although HF solutions are effective, these solutions are also more dangerous and tedious to handle. An alternative to F^−^ solutions that is easier to handle, but still efficient, is KF solutions. KF solutions are low in cost, simple, and biocompatible in addition to providing lower cytotoxicity levels. Pereda et al. has evaluated the effect of different KF concentrations on powder metallurgy Mg (Mg(PM)) [67]. The Mg powder (99.8%, 325 mesh) was cold-pressed up to 310 MPa, obtaining a Mg rod, which was cut into 1 cm diameter disks prior to mechanical polishing. The Mg (PM) samples were treated in 0.1 M and 1 M KF solutions from 1 hour to 168 hours (7 days). Results indicated the presence of KMgF_3_ cubic crystals in the protective coating. Electrochemical tests showed that 0.1 M KF pretreatment of the alloys exhibited higher corrosion resistance than 1 M KF pretreatment. Other acids such as phosphoric acid and sulfuric acid can also be used to increase surface bioactivity (i.e., in a mixed acid solution of 2% H_3_PO_4_ and H_2_SO_4_ at room temperature for 5-10 s) [62].

Tannic acid (C_76_H_52_O_46_) is an organic compound that can react with metal ions to form tannic acid-metal complexes. Zhu et al. performed electrodeposition of HAp onto magnesium alloys (AZ31) using tannic acid as the inducer follow by a study of the corrosion behavior of the coating in SBF solution for both treated and untreated samples [68]. Before the acid treatment, the samples were soaked in 1 M NaOH for 24 hr followed by heating at 150°C for 1 hr; after that, the samples were soaked and kept in tannic acid at 37°C for 9 hr. After the tannic acid treatment, the substrate was then immersed into a CaP solution at constant temperature (37°C) for 48 hr, CaP solution was replaced every 24 hr. The immersion test in SBF solution was done for the set of samples including bare magnesium alloys (AZ31), magnesium alloys treated tannic acid (TA/AZ31), bare magnesium alloys coated HAp (HA/AZ31), and treated magnesium alloys coated HAp (TA/HA/AZ31) before the surface analysis. The immersion test was performed for 7 days; during the experiment, hydrogen release was reported, and SBF solution was changed every 24 hr.

Before immersion in SBF solution, SEM results revealed that the surface of TA/AZ31 had a uniform structure with decreasing cracking compare to bare AZ31 surface. The HAp also grew thicker and more uniform on the surface of TA/HA/AZ31 than HA/AZ31 [68]. Therefore, tannic acid pretreatment not only decreased cracking on the surface of bare magnesium alloys but also promoted deposition of HAp onto the substrate. After soaking in SBF solution, TA/AZ31 showed less cracks and pits compared to the bare surface of AZ31; uniform, dense, and spherical particles formed on the TA/AZ31 surface. The TA/HA/AZ31 surface after soaking also had less cracks and pits, the surface self-healed after soaking in SBF solution by redeposition of CaP [68]. EDS was also performed on the surfaces of TA/AZ31 and TA/HA/AZ31; the results revealed a new layer on the surface of TA/AZ31 by detecting C (41.63%) and O (40.68%) with lower amount of Mg (17.69%). On the surface of TA/HA/AZ31, Ca and P were detected with the atomic ratio of Ca/P 1.62, which is very close to the ratio of hydroxyapatite (1.67) [68]. Corrosion testing was also performed for all samples; the value of R_p_, E_corr_, and I_corr_ is reported in Table 4, in which TA/HA/AZ31 appeared to have the best corrosion resistance compared to all others.

#### 2.3.3. Anodizing

Anodizing is an electrolytic oxidation process that creates a thick, durable, abrasion-resistant, and adherent film on the substrate. During anodization, the metal substrate serves as the anode of an electrical circuit producing a protective conversion coating on the surface. Song et al. anodized (commercial purity) CP-Mg coupons in a bath containing 1.6 wt% K_2_SiO_3_ + 1 wt% KOH, by applying a DC current density of 20 mA/cm^2^ for 30 minutes [60]. This process resulted in a ~4 *μ*m thick coating containing magnesium oxides/hydroxides and less than 30% silicon oxides/hydroxides. It should be noted that this anodized coating is nontoxic to the human body since there are essential traces of Si reported in mammals. The anodized magnesium substrate was submerged in SBF solution for one month and no hydrogen evolution was detected, showing the corrosion resistant quality of the anodized coating and its success in delaying the biodegradation of the substrate. For high efficiency, anodizing in an alkaline electrolyte solution is preferred as well as controlling the temperature [69]. The thickness of the anodic oxide layer also decreases when the temperature of the electrolyte solution increases.

#### 2.3.4. Microarc Oxidation (MAO)

Microarc oxidation (MAO) has recently been used to increase the oxide layer on substrates. While similar to anodic oxidation, it is an electrochemical process that uses higher potentials than anodic oxidation to induce discharges/plasma that modify the structure of the oxide layer. The higher applied potential generates an electric field above the breakdown potential creating a crystallization process that would not occur in a milder environment (anodization). Possible reactions that can occur during MAO of Mg or Mg alloys are indicated in (16)-(21) [70]:

(16) Mg→Mg2++2e−

(17) 4OH−→O2↑+ 2H2O+4e−

(18) 2H2O→2H2↑+ O2↑

(19) Mg2++2OH−→MgOH2↓

(20) MgOH2→MgO↓+ H2O

(21) 2Mg+O2→2MgO↓

In one study, MAO was conducted on a Mg-Ca (1 wt.%) alloy ingot at a fixed applied voltage in the range of 300-400 V for 10 min [71]. The pore size and thickness of the MAO layer increased with increasing applied voltage. The optimal voltage was found to be at 360 V for long-term corrosion protection. The MAO layer consisted of MgO and Mg_2_SiO_4_ phases formed beside the *α*-Mg phase. The rate of hydrogen evolution (0.007 mL cm^−2^ day^−1^) was most reduced when 360 V was applied as opposed to when 300 V was applied (0.108 mL cm^−2^ day^−1^). The pH of cultured medium reduced significantly for the treated substrate compared to the untreated Mg alloy, pH 9 and 11, respectively, due to greatly reduced Mg dissolution. Improvement in cell adhesion and proliferation was also observed. Similar and effective results can be utilized in other biomedical magnesium alloys, e.g., AZ91 and AZ91D [72, 73].

### 2.4. CoCrMo Alloy

Cobalt-based alloys can be extensively used due to their excellent corrosion resistance, biocompatibility, and strength. With the addition of molybdenum to these alloys, an orthopedic implant material has emerged and demonstrates a remarkable level of versatility and durability [74]. Recently, CoCrMo alloys have been sufficiently researched as an alternative to other biomedical alloys (i.e., metal-on-metal hip resurfacing joints) due to their superior strength and robust surface hardness, which increases resistant to wear in vivo [18, 75–79]. The corrosion resistance of CoCrMo alloys is due to the protective layer that spontaneously forms on the surface, inhibiting corrosion and the release of metal ions. This protective layer consists of oxides, including Cr_2_O_3_ and its other oxidation states, Co-oxides, and Mo-oxides [80, 81]. Surface pretreatments prevent the release of harmful metal ions (i.e., Cr^6+^) in vivo, producing desirable properties on the surface of the material. There are fewer studies investigating the effect of surface pretreatment on CoCrMo alloys, with the research still emerging, compared to titanium alloys.

#### 2.4.1. Acidic Pretreatment

Polishing and chemical etching with acids are the most common types of surface pretreatments for CoCrMo alloys. This cleaning process smooths the surface roughness, which reduces friction and increases the adhesion strength between the metal surface and HAp film [80]. The etching efficiency of surface pretreatment varies with the types of acid used, immersion time, and temperature.

**Figure 8:**
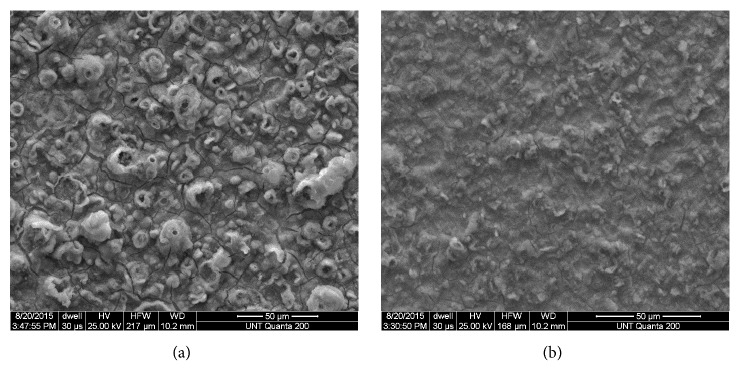
SEM images of HAp coatings electrochemically deposited onto CoCrMo alloy with (a) 0 mM aspartic acid and (b) 10 mM aspartic acid. (Courtesy I. Coskun and T.D. Golden, 2018).

CoCrMo alloys have been etched in combinations of different acids including HCl, HNO_3_, HF, and acetic acid [80]. Coşkun et al. [18, 77, 79] used commercially provided CoCrMo dental alloy (Co-58.3%, Cr-32%, Mo-6.5%, W-1.5%, and Si-1.0%) as a substrate. After polishing, the substrates were degreased then pretreated with 1 M HCl and then 10% HF solution. Addition of amino acids, such as aspartic acid during electrodeposition of HAp also affected the hydrogen evolution at the surface of the substrate.  Figure 8 shows the SEM of the HAp-coated substrate for an untreated and treated (10 mM aspartic acid addition) CoCrMo alloy. For the untreated surface, H_2_ gas formation disrupted the coating process and produced pores and cracks. The addition of 10 mM aspartic acid represses hydrogen evolution and as a result produces adherent smooth coatings and significant crystal growth of HAp on the substrate (Figure 8).

There is also improvement in the corrosion performance of the CoCrMo samples in SBF solution with the addition of aspartic acid.  Table 5 lists the E_corr_ and i_corr_ values obtained from potentiodynamic polarization experiments for the treated and untreated samples. The highest corrosion resistance was observed for HAp coatings deposited from 10 mM aspartic acid containing solutions. An anodic shift in E_corr_ values from approximately -0.480 V vs SCE for the untreated sample to -0.300 V vs SCE for the treated substrates is observed indicating a more passive nature and a better corrosion resistance for coatings. Also the corrosion rate (i_corr_) decreased for the treated CoCrMo substrates.

Hamtaiepour et al. [80] used several different acid pretreatments, in combination with heat, prior to coating the surface with HAp via physical vapor deposition. The surface roughness of the substrate was measured after each treatment and pits in the substrate were examined by SEM. The time and temperatures of the acid pretreatments 1 (HF + HNO_3_ + Ethanol) and 2 (HCl + HNO_3_ + acetic acid + H_2_O) had the most significant impact on the surface morphology. Micropits started to form in 30 seconds and in 240 seconds at 50°C using acid bath 2 and 1, respectively. The micropits, produced after etching the surface of CoCrMo alloy, were hypothesized to increase the adhesion strength of the coating material without sacrificing the smoothness of the substrate [80]. In another study, Izman et al. [81] used two methods of pretreatments, chemical and mechanical, to obtain different sets of surface roughness. The chemical method involved pickling CoCrMo alloy (ASTM F1537) disks in 50 mL of HNO_3_ (65%) + 150 mL HCl (37%) and then ultrasonically cleaning in acetone for 30 minutes. The mechanical method involved polishing the disks to a mirror finish using SiC and diamond paste grit. The chemical and mechanical pretreated samples were then oxidized in a muffle furnace at 1160°C for three hours under atmospheric condition and cooled inside the furnace for four hours. Several types of oxides and carbides were detected in the chemically treated samples such as Cr_23_C_6_, CoCr_2_O_4_, Cr_2_O_3_, CoO, and MoC. Among these, Cr_23_C_6_ was the dominant product observed when using mechanical methods as well as CoCr_2_O_4_. Results also indicated that mechanically treated samples had 12% higher hardness than chemically treated, where a higher amount of carbide was formed using mechanical treatments. This is most likely due to the diamond paste being trapped in the roughness valleys which react with the metal matrix to later form carbides during the oxidation process. Different types and combination of acids, the amount of time etched, and temperature of the acid bath greatly affect the surface morphology of the CoCrMo alloy surface. The aforementioned studies illustrated the effect of using different parameters, but much research still needs to be done to test the in vivo quality of pretreated CoCrMo alloy substrates coated with HAp.

#### 2.4.2. ECAD Pretreatment

Using electrochemically assisted deposition (ECAD) as a pretreatment has shown to increase the adhesion strength between the HAp film and the substrate as well as enhance the capability of HAp formation [82]. This process has also been used for other metallic implants such as titanium and tantalum alloys. During ECAD, an electric current is applied to two electrodes, which are immersed in an electrolyte containing calcium and phosphate. At the cathodic implant substrate, CaP species are then deposited. The electrochemical reactions that occur near the surface of the cathode include, reduction of water and dissolved oxygen (shown in (22)-(24)) [82]:

(22) 2H2O+2e−→H2+2OH−

(23) 2H3O++2e−→H2+H2O

(24) O2+H3O++4e−→3OH−

From these reactions, the pH increases locally at the cathode's surface where nucleation of CaP on the substrate is induced and a film is formed. After ECAD pretreatment, an alkaline treatment is then followed to enhance the adhesion of the film to the substrate. There are many factors that can alter the surface morphology of the film including the deposition current, duration time, and the contents of the electrolyte solution (addition of oxidants and organic species). Wang et al. [82] used CoCrMo disks (ASTM F1537) as substrates. The disks went through two pretreatments and a chemical post treatment. The samples were first cleaned in concentrated H_2_SO_4_ for 1 minute to remove any impurities as the first pretreatment. The disk was then ECAD pretreated via a three-electrode electrochemical cell in a supersaturated solution containing calcium and phosphate as the electrolyte. The electrolyte contained NaCl, CaCl_2_, MgCl_2_·H_2_O, NaHCO_3_, Na_2_HPO_4_·2H_2_O, and 1 M HCl; and adjusted to pH 6. A constant pulsed potential of -1.5 V with respect to saturated calomel electrode at ambient temperature, for 10 min, was applied. The ECAD-pretreated CoCrMo alloy produced a light yellow color. Results indicated that using ECAD as a pretreatment enhances the formation of HAp coating due to the formation of a thin 200 nm layer of calcium phosphate on the surface of the substrate. This is due to the localized pH increase at the cathode, facilitating the precipitation of calcium and phosphate on the surface.

#### 2.4.3. Oxidation Pretreatment

Studies have shown for other metals such as Ti alloys that having an intermediate oxide layer enhances the adherence of the HAp coating to the substrate [83, 84]. The use of oxidation techniques to create the oxide layer on the surface of CrCoMo has been shown by Ayu et al. [85]. This technique was used to lower the cost and shorten the process time for CoCrMo alloys. Before the oxidation pretreatment, the substrate was ultra-sonicated with acetone for 30 minutes followed by complete drying using a stream of compressed air. The oxide layer was produced by heating at 1050°C for 3 hr under atmosphere and left to cool for 4 hr. This process created a layer of Cr_2_O_3_, confirmed by SEM. HAp coatings were made using a dip-coating method both with and without oxide layer substrates. The substrates were immersed in a HAp slurry and withdrawn at the rate of 200 mm/min, the process was repeated 4 times to complete a coating. Eventually, the coatings were sintered at 550, 650, and 750°C for 1 hr. As a result, the morphology of CoCrMo surface after the oxidation pretreatment appeared to have a higher roughness (1 *μ*m) compare to the untreated substrate (0.1 *μ*m). This was explained by the formation of increasing size Cr_2_O_3_ particles of 100 to 700 nm, which led to creating the massive voids in the layers. The cross section of these samples was also analyzed, which showed that the outer layer (HAp coating) was more compact but thinner (12.73 *μ*m) than the inner layer (Cr_2_O_3_) (51.03 *μ*m). SEM of the HAp coating for both treated and untreated substrates was also performed, showing that the coating on the untreated substrates had more cracks which were larger than on the coating of the treated substrates. Ayu concluded that the higher the sintering temperature, the smaller and less cracking seen on the coating surfaces. It was also found that, as the temperature increased, a thinner HAp coating resulted and a thicker oxide layer [85].

## 3. Conclusions

As covered in this review, there are numerous studies on substrate pretreatment to induce hydroxyapatite formation, and improve bioactivity and biocompatibility for metals and metal alloys.  Table 6 compiles the surface activation techniques discussed in this review.

Surface activation techniques can enhance several properties by forming a strong barrier between the metal substrate and body fluid and increasing corrosion resistance [86]. By pairing a surface pretreatment with heat treatment, some unwanted oxides can be removed while other oxides that promote protection are initiated [87]. The standard Gibb's free energy change (∆*G*_1_^0^) values for many metal oxides can be calculated from specific heat data or using thermodynamic modeling software in order to derive temperature dependence of equilibrium oxygen partial pressure [88, 89]. The decomposition of more stable oxides is facilitated by lowering the oxygen partial pressure by several orders of magnitude. These partial pressures and high temperatures can be achieved through a vacuum furnace and can be used as pretreatment protocols.

The applied surface treatments remove a majority of inclusions that initiate pitting corrosion. For example, stainless steel and chloride ions initiate pit growth by increasing the acidity of the electrolyte (see (25)).

(25) $$\begin{array}{c} {FeCl}_{2}+2H_{2}O=Fe{\left(\;OH\;\right)}_{2}+2HCl \end{array}$$

The pit areas are positively charged, attracting chloride ions, forming 2 mols of HCl for every one mole of iron. The SS surface then becomes fouled due to the Fe(OH)_2_ by-products formed around each pitting zone, creating a barrier between the solution and the substrate. Under some conditions, the release of iron to nearby tissue produced by localized corrosion can cause fibrosis around the implant [90]. Through surface activation of SS, MnS inclusions and free iron ions are removed as well as passivating the surface by forming chromium oxide and enriching the Mo content. Acid pretreatment for stainless steel substrates not only improves adhesion but has been shown to reduce grain size of electrodeposited nanocomposite hydroxyapatite coatings [91].

Surface activation of titanium and SS achieves similar features when pretreated in an alkaline solution [92]. Both substrates obtain a hydrated gel layer that later induces apatite formation, illustrating the dissolution of metal oxygen passive layer to form a metal hydroxide layer. The alkali-treated substrates obtained a passive layer consisting of sodium titanate and sodium chromate for titanium and SS substrates, respectively. The thickness of the oxide layer was highest when titanium was treated in 5 N NaOH and SS in 20 N NaOH. Researchers have also indicated a better corrosion resistance when a double- or multilayer was applied onto implants, such as the chromium oxide and sodium chromium oxide layer that can be produced on the surface of 316L SS prior to coating with HAp [49]. The cleanliness of the substrate is also crucial prior to pretreatment. The substrates need to be degreased and polished in order for the surface activation to be effective. Bodily fluids contain chloride ions that will aggressively target metals and alloys introducing pitting corrosion [49].

As covered in this review the most common metals and alloys used for biomedical application are Ti and its alloys, 316L SS, and CoCrMo. These materials primary applications have been in the orthopedic field for joint replacements and dental implants [93]. Other materials such as Mg and its alloys have been studied as a possible substitute substrate due to its high strength-to-weight ratio and similar properties to bone. The biodegradable property of magnesium metal is a key advantage, negating the need for a second operation for implant removal. Surface modification of Mg alloys is also important to minimize corrosion during use and encourage osseointegration and biocompatibility. For example, electrochemical anodic oxidation has been utilized to initiate thick and uniform metal oxide layers [36]. Building a coating with a MAO inner layer and a HAp outer layer can enhance corrosion and improve bioactivity and bonding strength in Mg alloys [94]. A recent study of only microarc oxidation pretreatment examined the relationship between porosity, thickness, microhardness, and surface morphology as a function of microarc parameters [95]. Current frequency of the microarc technique affected the porosity and the pore diameter of the resulting films. Lower porosity and better continuity of the films improved the corrosion resistance of the films. Alkaline pretreatment of Mg alloy substrates has also shown to enhance corrosion resistance and bonding strength of the deposited bioapatite [96]. The parameters of this technique can be easily manipulated in order to finely tune the oxide layer. The alkali pretreatment produces a Mg(OH)_2_ thin film that tightly bonds to the substrate. This layer formed by alkali and thermal pretreatment increases the bonding strength with the HAp coating.

The efficiency of the implant is not limited to only surface activation techniques, but the stability and long-term performance of the HAp-coated implant are also governed by the quality of the HAp coating itself. HAp has similar chemical composition to bone and teeth and also improves the corrosion resistance of the material. Characteristics of HAp such as purity, crystallinity, Ca/P ratio, microstructure, porosity, thickness, and of course surface properties of the metallic substrate are all features that greatly influence the quality and performance of the coated implant [33]. Although there are many ways to coat HAp onto substrates, electrodeposition has several advantages as a technique. Other techniques such as growing hydroxyapatite through immersion in SBF solution can take days or weeks and the extremely high heat from plasma spraying causes some decomposition to soluble calcium phosphate compounds due to the thermal instability of hydroxyapatite [97]. Electrodepositing hydroxyapatite onto the metallic substrates gives the constructive ability to control the crystal growth and thickness of the film [98]. With this control, the parameters, morphology, and size can be easily altered and refined. A strong barrier between the coated substrate and environmental body fluids will increase the lifespan of the implant, decreasing the amount of metals leeching in vivo. The enhancement of hydroxyapatite adhesion via surface activation techniques onto a metallic substrate is necessary for implant applications, especially for corrosion resistance to lower degradation rates.

Future trends will show that new and improved pretreatment routes will continue to be developed for biocompatible implants. As an example, laser-induced pretreatment has recently been developed to improve the ingrowth of implants into the surrounding bone. By increasing the surface area of the substrate, biocompatibility can be improved. In one study, a laser-based technique was used to generate nanostructures with cavities between 20–30 nm on titanium alloys [99]. However further studies are needed to determine the optimal surface roughness, size, and pattern of micro- and nanostructures of implants to increase biological and mechanical stability. Controlled nano/micropattering of the substrate surfaces should affect the properties of the bioapatite layer. Future studies are needed to relate the nanostructures on the substrate surfaces with ensuing properties of the deposited coatings. Another trend may find that combining the pretreatment and deposition steps yields faster and improved results. A recent study did in situ synthesis of HAp/TiO_2_ coatings on titanium substrates by combining anaphoretic deposition of HAp and simultaneous anodization of titanium [100]. The composite coatings produced were highly adherent with HAp nanocrystals incorporated into the oxide film. Similar combination techniques may hold promise for all the biocompatible substrates.

## Figures and Tables

**Table 1:** Surface properties obtained from immersing Ti substrates in various H_2_O_2_ baths.

Procedure	Characteristic Results
200 mL of 35% H_2_O_2_ at 60°C for 2 hrs, then 48 hrs at R.T. [27]	(i) Dense and amorphous HAp composite layer (ii) Similar characteristics to NaOH pretreatment (iii) Induced fast formation of uniform HA coating

10 mL of 5 M H_2_O_2_ at 60°C for 24 hrs. [32]	(i) Produced thicker and more porous oxide layer (~0.06 *μ*m) (ii) Provided more favorable sites for CaP nucleation (iii) Formation of basic TiOH groups was accelerated

5M H_2_O_2_/0.1 M HNO_3_ (pH 7) at 80°C for 20 min. [31]	(i) Anatase-type TiO_2_ oxide layer with very low crystallinity (ii) Obtained sponge-like morphology (iii) Homogenous and uniform formation of HAp clusters

**Table 2:** Various electrolyte solutions and applied potentials used for anodizing Ti substrates [39].

Sample	Electrolyte solution	Applied potential (V)	Results
1	1 wt% HF	60	Dot-like structures from fast dissolution of oxide layer

2	1 M H_3_PO_4_+ 1 wt% HF	60	Nanopowder granules on dot-like structures

3	5 M H_3_PO_4_+ 1 wt% HF	60	Nanopowders

4	10 M H_3_PO_4_+ 1 wt% HF	60	Nanopowders + Nanotubes

5	1 M H_3_PO_4_	60	Cracking of barrier oxide layer

6	1 M H_3_PO_4_	200	Microporous structure

7	1 M H_3_PO_4_+ 1 wt% HF	20	Nanotubes

**Table 3:** Average rate of hydrogen evolution for various Mg alloys [60].

Substrate	Average rate of hydrogen evolution (mL/cm^2^/day)
CP-Mg (Commercial Purity)	26

ZE41 (~4 wt% Zn, ~1 wt% RE, 0.4-1 wt% Zr, ~0.005 wt% Fe, ~0.1 wt% Cu and ~0.01 wt% Ni)	1.502

HP-Mg (High Purity)	0.008

Mg1.0Zn (~1.0 wt% Zn, ~0.02 wt% Fe, <0.002 wt% Cu and <0.001 wt% Ni)	0.280

AZ91 (~9 wt% Al, ~1 wt% Zn, ~0.005 wt% Fe, <0.002 wt% Cu, and <0.002 wt% Ni)	0.068

Mg2Zn0.2Mn (~2 wt% Zn, ~0.2 wt% Mn, 0.0013 wt% Fe, <0.002 wt% Cu, <0.001 wt% Ni)	0.012

**Table 4:** The polarization resistance (R_p_), corrosion potential (E_corr_), and corrosion current density (I_corr_) of the AZ31, TA/AZ31, HA/AZ31, and TA/HA/AZ31 samples in SBF at 37°C [68].

Samples	E_corr_ (V)	I_corr_ (A/cm^2^)	R_p_ (Ω cm^2^)
AZ31	-1.462 ± 0.006	(4.8978 ± 0.2455) x 10^−6^	6203

TA/AZ31	-1.416 ± 0.011	(3.7334 ± 0.3461) x 10^−6^	25,634

HA/AZ31	-1.391 ± 0.007	(3.9337 ± 0.2465) x 10^−7^	-* *-* *-* *-* *-

TA/HA/AZ31	-1.304 ± 0.006	(5.6494 ± 0.3187) x 10^−8^	63,637

**Table 5:** Potentiodynamic polarization values for acid pretreatment of CoCrMo alloys (Courtesy of I. Coskun and T.D. Golden, 2018).

Aspartic acid addition (mM)	E_corr_ (V vs SCE)	I_corr_ (A/cm^2^)
0	-0.480	1.0 × 10^−8^

4	-0.465	1.1 × 10^−8^

8	-0.299	2.5 × 10^−8^

10	-0.310	7.9 × 10^−9^

**Table 6:** Summary of pretreatments and results for different biocompatible substrates.

Substrate	Pre-treatment	Surface properties
Ti and its alloys	Alkaline	Hydrated Ti oxide gel layer
	Acidic	Removes free metal, increases metal oxide layer
	H_2_O_2_	Forms titanium dioxide and titanium hydroxide
	Anodizing	Titanium dioxide nanotube layer, increases natural oxide layer
	Sandblasting	Increases roughness and surface area, activates surface.

Stainless Steel	Alkaline	Hydrous metal oxide layer
	Acidic	Removes MnS inclusions, creates Cr oxide layer, enriches Mo (noble element)
	Electron beam	Removes MnS inclusions, melted surface forms strong interfacial bond with substrate

Mg and its alloys	Alkaline	Increases surface area and roughness
	Acidic	KMgF_3_ cubic crystals in the protective coating
	Anodizing	Creates thick and porous oxide layer
	Micro-arc oxidation	Creates thick and porous oxide layer

CoCrMo alloy	Acidic	Creates oxide layer, including CoCr_2_O_4_, Cr_2_O_3_, Co oxides, and Mo oxides.
	ECAD	Increases adhesion strength between the HAp film and substrate as well as enhance the capability of HAp formation.
